# Results of Functional Treatment of Epi-Metaphyseal Fractures of the Base of the Fifth Metatarsal

**DOI:** 10.1177/1071100720907391

**Published:** 2020-02-26

**Authors:** Sebastian F. Baumbach, Marcel Urresti-Gundlach, Wolfgang Böcker, J. Turner Vosseller, Hans Polzer

**Affiliations:** 1 Department of General, Trauma and Reconstructive Surgery, University Hospital, LMU Munich, Munich, Germany; 2 Department of Orthopaedic Surgery, Columbia University Medical Center, New York, NY, USA

**Keywords:** fifth metatarsal fracture, Lawrence and Botte, functional treatment, fracture characteristics, outcome

## Abstract

**Background:**

Fractures of the fifth metatarsal base (5th MT) are common foot injuries, but their treatment remains a subject of debate. The aim was to assess the midterm outcome of functionally treated epi-metaphyseal fractures (Lawrence and Botte types I and II) of the 5th MT.

**Methods:**

This study was a longitudinal retrospective database study with prospective follow-up. Included were all patients with an acute, isolated fracture to the 5th MT base (types I and II). All patients were treated functionally: weightbearing as tolerated without immobilization. Fracture types and fracture characteristics (displacement <2 mm/>2 mm, articular involvement, number of fragments) were assessed retrospectively. Patient-reported outcome measures (PROMs) including the visual analog scale for foot and ankle (VAS FA) and the quality-of-life score (QoL) SF-12 were collected prospectively at 2- and 5-year follow-up. Out of 95 patients, 43 patients (45%) were included with a median follow-up of 5.7 (1.5) years.

**Results:**

For both the VAS FA and SF-12, excellent scores were observed. For 30 patients (77%), longitudinal 2- and 5-year follow-up was available. No significant longitudinal changes could be observed for the VAS FA and SF-12. For both time points, neither fracture type nor characteristics significantly influenced any outcome parameter assessed.

**Conclusion:**

Functional treatment by full weightbearing and free range of motion led to excellent 5-year results for both type I and II fractures. Neither fracture location nor characteristics had a significant influence on the 5-year PROMs.

**Level of Evidence:**

Level III, comparative study.

Fractures to the base of the fifth metatarsal (5th MT) are among the most common injuries of the foot.^14^ The treatment of these injuries is a matter of significant debate, especially for type I and II fractures according to Lawrence and Botte,^16^ which account for more than 90% of all fractures of the base of the 5th MT.^14^ The Lawrence and Botte classification is outlined in Figure 1.

**Figure 1.**
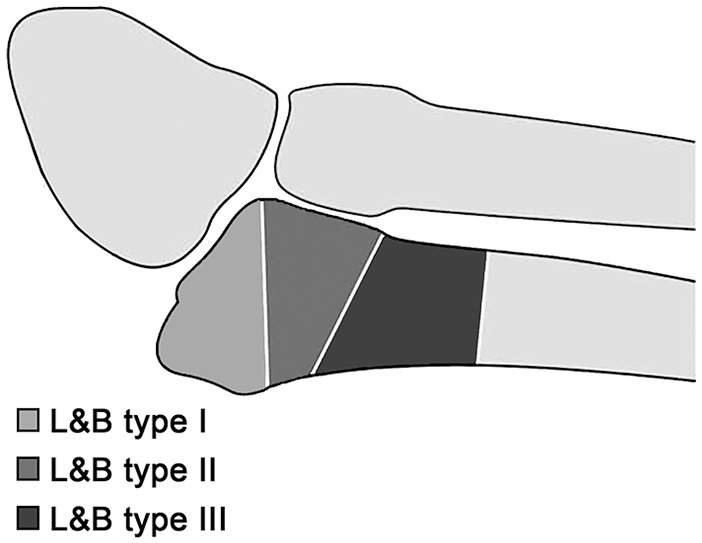
Schematic illustration of the Lawrence and Botte^16^ classification. Adapted from Baumbach et al.^3^ Publication covered by the CC BY 4.0.

Surgical indications for type I fractures have varied in the literature, but they have included some of the following: displacement of ≥2 mm, involvement of more than 30% of the articular surface, or comminution.^33^ These recommendations are not based on evidence but rather reflect single authors’ opinions.^4,11,16,19,34^ Few studies have actually analyzed the influence of those fracture characteristics on patient-reported outcome.^3,9,29^ Unfortunately, these studies presented only short-term follow-up (20 weeks to 2 years). Consequently, we are still missing mid- and long-term results for displaced, intra-articular, and comminuted type I fractures.

Studies on type II fractures are rare because of the inconsistent use of the term *Jones fracture*, which has been used synonymously for both type II and III fractures.^2,3,8,22^ While there is strong evidence supporting operative treatment for type III fractures,^18^ recommendations for type II fractures vary from immobilization and nonweightbearing to operative treatment.^8,16,19,20^ Some authors, on the other hand, have hypothesized that functional treatment can lead to favorable results.^6,9,15^ Again, mid- to long-term results are missing.

We have treated all patients with an acute epi-metaphyseal fracture (type I or II) functionally with full weightbearing and free range of motion since 2012. We previously published promising 2-year results following functional treatment of any epi-metaphyseal proximal 5th MT fracture.^3^ The aim of the current study was to assess the midterm outcome for functionally treated epi-metaphyseal fractures of the 5th MT bone (types I and II).

## Methods

This was a retrospective, longitudinal database study, with a prospective 5-year follow-up. As all type I and II fractures were included and no intervention was applied, this study can be considered a natural history study. The study was approved by the local ethics committee. The 2-year patient-rated outcome has been published previously.^3^ The detailed patient selection process for the 5th MT registry has been described previously.^3^ In summary, the database included 95 patients meeting the inclusion criteria (acute, isolated epi-metaphyseal fracture [type I or II], >17 years old, functional treatment). Of those, 39 (41%) could be followed up after 2 years.^3^ For the current study, all 95 patients were contacted again.

All patients who sustained an acute, isolated epi-metaphyseal fracture (types I and II) were treated functionally by full weightbearing as tolerated, as outlined previously.^3,22^ Immobilization was performed only if requested by the patient but for a maximum of 14 days. No routine radiological follow-up was performed. Radiological follow-up was performed only if patients were symptomatic after 6 weeks. This treatment regimen was applied to all patients, independent of the fracture characteristics, namely, number of fragments, displacement, or articular involvement.

### Data Assessment

Demographic data, fracture location per Lawrence and Botte^16^ (type I or II) and fracture characteristics (number of fragments [binary; 2 or multiple], displacement [binary; ≤2 mm, >2 mm], intra-articular involvement [binary]) were assessed retrospectively. Patient-rated outcome measures (PROMs) were assessed prospectively, namely, the visual analog scale for foot and ankle (VAS FA)^23^ and the SF-12. The VAS FA is a 20-item foot and ankle outcome measure, with an overall score (Overall) and 3 subscales (Pain, Function, Other).^23,28^ Scores range from 0 to 100 points. Published reference values for healthy controls are 86 to 100 for “Overall,” 82 to 100 for “Pain,” 87 to 100 for “Function,” and 68 to 83 for “Other.”^28^ The SF-12, one of the most commonly applied general quality-of-life tools, has 2 meta-scores: the Physical Component Summary (PCS) and Mental Component Summary (MCS). Values of 50 are representative for a healthy population, with higher scores representing a better outcome. The primary aim of this study was to assess the VAS FA after an average of 5 years following functional treatment of any type I and II fracture. Secondary outcome parameters were the quality-of-life score (SF-12) and the identification of factors influencing the patient-rated outcome after 5 years as well as the comparison of the longitudinal follow-up after 2 and 5 years. The 2-year follow-up cohort has been published previously.^3^

**Figure 2.**
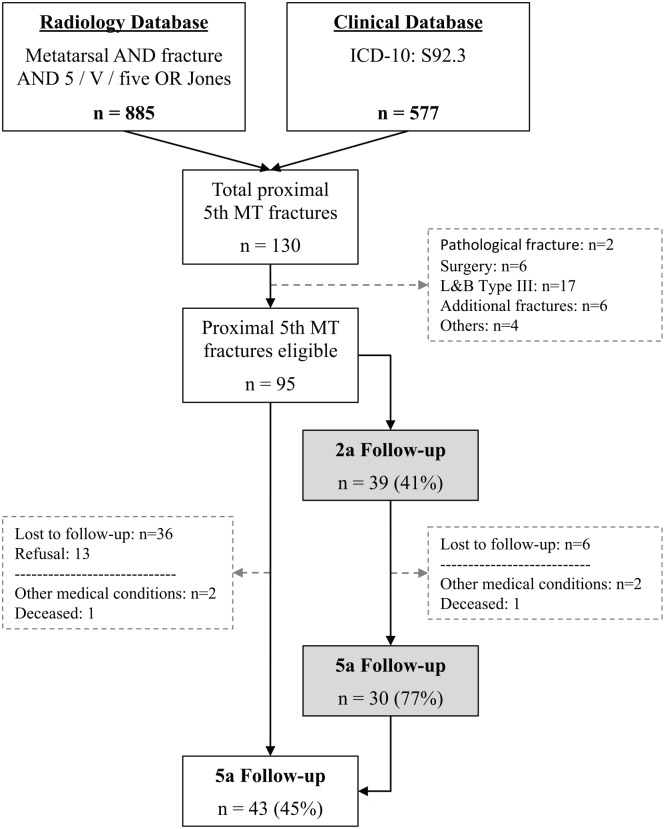
Patient selection flowchart. Gray fields: patients with a longitudinal 2- and 5-year follow-up. L&B, Lawrence and Botte classification; n, number of patients; 5th MT, fracture to the proximal fifth metatarsal bone.

Out of the 95 patients included in the database, 43 (45%) patients were available for a 5-year follow-up. Thirty patients had a prospective 2- and 5-year follow-up. The patient selection is depicted in Figure 2. The demographics of the 5-year cohort, including a comparison between the longitudinal 2-year cohort and the additional 13 patients recruited from the original database, are shown in Table 1. No significant differences were found for the demographics and fracture characteristics between these cohorts.

**Table 1.** Demographics and Fracture Characteristics of the Different Patient Cohorts.

Characteristic	5-Year FU Cohort	2-Year FU Cohort	Additional Recruited Patients 5-Year Cohort	*P* Value
Number of patients	43	30	13	
Age at trauma, median (interquartile range), y	43 (23)	41 (22)	52 (21)	.265
Sex, % female	61	57	69	.513
Side, % left	44	47	39	.743
L&B type I, %	63	60	69	.735
Displacement (≥2 mm), %	30	33	23	.720
Intra-articular, %	79	73	92	.237
Multifragmentary, %	47	40	62	.318

Abbreviations: FU, follow-up; L&B, Lawrence and Botte classification.

### Statistics

The Kolmogorov-Smirnov normality test on the current VAS FA Overall (5a) revealed nonnormal distribution (*P* < .001). Consequently, nonparametric testing was applied, including standard descriptive statistics, Fisher exact test, Mann-Whitney *U* test, related-samples Wilcoxon signed rank test, and Spearman’s correlation. The level of significance was set at *P* ≤ .05 for the primary outcome. A Bonferroni alpha-level correction was performed for the secondary outcome, setting the level of significance to *P* = .007. Data are presented as median (interquartile range), if not stated differently. Statistics were computed using SPSS (Version 25; SPSS, Inc, an IBM Company, Chicago, Illinois).

## Results

### Patient-Rated Outcome at 5-Year Follow-up

**Figure 3.**
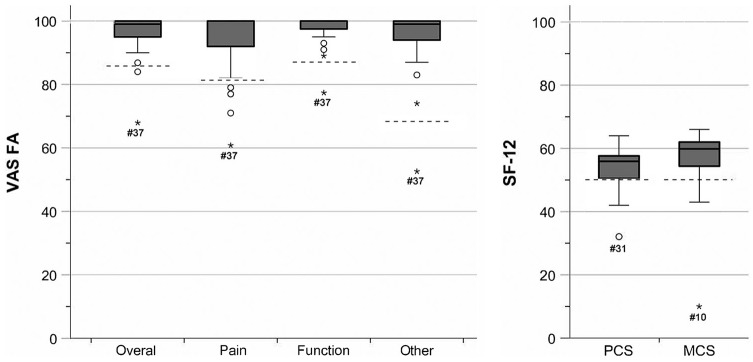
Visual analog scale for foot and ankle (VAS FA) and quality-of-life score (SF-12) at 5-year follow-up. The dashed lines depict the lower limit of the reference values of healthy individuals; for the VAS FA scores: Overall: 86 to 100; Pain: 82 to 100; Function: 87 to 100; Other: 68 to 83.^28^ The Physical Component Summary (PCS) and Mental Component Summary (MCS) of the SF-12 for a healthy reference population are 50 points. *Indicates outliers.

The median follow-up for the 5-year follow-up cohort was 5.7 (1.6) years. The results for the VAS FA and SF-12 are depicted in Figure 3. In total, 3 outliers with a conspicuously poor result were observed. For the VAS FA, patient 37 (age 43 years, female, fracture: type II, not displaced, 2 fragments, intra-articular involvement) presented the lowest scores for all domains (Overall, Pain, Function, Other) but above-average scores for the SF-12 (PCS = 51; MCS = 58). For the PCS subscale of the SF-12, patient 31 (age 66 years, male, fracture: type I, not displaced, multifragmentary, intra-articular involvement) scored inferior (32) while the results of the VAS FA did not show any foot and ankle–related problems (100 on all subscales). Finally, patient 10 (age 60 years, female, fracture: type II, displaced, multifragmentary, extra-articular) scored considerably inferior on the SF-12 MCS (2 years = 25; 5 years = 10) with no general physical (PCS = 64) or foot and ankle–related impairment (VAS FA: Overall = 95, Pain = 100, Function = 99, Other = 83).

The influence of various parameters (ie, demographics, fracture type, and characteristics of the 5-year PROM) was evaluated (Table 2). None of the parameters assessed had a significant influence on any PROM.

**Table 2.** Influence of Demographics and Fracture Characteristics on the 5-Year Follow-up.

Characteristic	VAS FA Overall	VAS FA Pain	VAS FA Function	VAS FA Other	SF-12 PCS	SF-12 MCS
Age	*r* = −0.154 *P* = .324	*r* = −0.016 *P* = .902	*r* = −0.190 *P* = .223	*r* = −0.231 *P* = .136	*r* = −0.244 *P* = .116	*r* = −0.096 *P* = .539
Sex	*P* = .074	*P* = .102	*P* = .157	*P* = .107	*P* = .200	*P* = .681
Side	*P* = .980	*P* = .989	*P* = .723	*P* = .364	*P* = .516	*P* = .873
L&B type	*P* = .011	*P* = .029	*P* = .041	*P* = .044	*P* = .134	*P* = .930
Displacement	*P* = .193	*P* = .667	*P* = .116	*P* = .557	*P* = .522	*P* = .276
Intra-articular involvement	*P* = .714	*P* = .826	*P* = .942	*P* = .268	*P* = .895	*P* = .649
No. of fragments	*P* = .287	*P* = .128	*P* = .675	*P* = .308	*P* = .550	*P* = .550

Abbreviations: L&B, Lawrence and Botte classification; MCS, Mental Component Summary; PCS, Physical Component Summary; SF-12, quality-of-life score; VAS FA, visual analog scale for foot and ankle.

Similar to the results after 2 years,^3^ fracture characteristics were compared per the Lawrence and Botte classification (type I vs type II) for the 5-year follow-up cohort. Neither displacement (*P* = 1), intra-articular involvement (*P* = .008), nor number of fragments (*P* = .761) differed significantly between the 2 fracture types (I vs II). Furthermore, age (*P* = .339/*P* = .093/*P* = .385/*P* = .050), sex (*P* = .052/*P* = 1/*P* = 1/*P* = 1), and side (*P* = 1/*P* = .509/*P* = .708/*P* = .760) did not differ for any fracture characteristic (Lawrence and Botte/displacement/articular involvement/number of fragments).

### Patient-Rated Outcome—Longitudinal (2 Years vs 5 Years)

**Figure 4.**
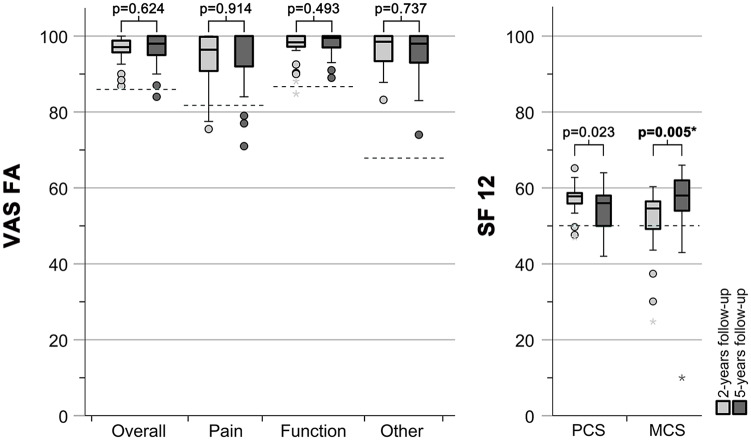
Boxplots illustrating the visual analog scale for foot and ankle (VAS FA) and quality-of-life score (SF-12) for the 2- and 5-year follow-up. The dashed lines depict the lower limit of the reference values of healthy individuals; for the VAS FA scores: Overall: 86 to 100; Pain: 82 to 100; Function: 87 to 100; Other: 68 to 83.^28^ The Physical Component Summary (PCS) and Mental Component Summary (MCS) for the SF-12 of a healthy population are 50 points. *Indicates outliers.

For 30 patients, a longitudinal outcome could be assessed at 2 different time points: 1.9 (1.4) and 5.0 (1.5) years after trauma. Figure 4 illustrates the outcome for the VAS FA and SF-12 comparing the 2- to 5-year follow-up, including the lower limit for the reference values of healthy individuals. Again, no significant differences between the 2- and 5-year follow-up were observed except a significant improvement of the MCS by 4 (10) to 58 (9; *P* = .005) points. Patient and injury characteristics were assessed to see whether any parameter had a significant influence on the difference (Δ) between the 2- and 5-year PROM follow-up (Table 3). None of the evaluated parameters had any influence on the change in PROMs over time.

**Table 3.** Influence of Demographics and Fracture Characteristics on the 5-Year Follow-up.

Characteristic	Δ VAS FA Overall	Δ VAS FA Pain	Δ VAS FA Function	Δ VAS FA Other	Δ SF-12 PCS	Δ SF-12 MCS
Age	*r* = 0.196 *P* = .300	*r* = 0.133 *P* = .483	*r* = 0.244 *P* = .193	*r* = 0.308 *P* = .098	*r* = 0.206 *P* = .275	*r* = 0.187 *P* = .323
Sex	*P* = .245	*P* = .053	*P* = .837	*P* = .263	*P* = .133	*P* = .483
Side	*P* = .608	*P* = .101	*P* = .275	*P* = .608	*P* = .728	*P* = .637
L&B type	*P* = .415	*P* = .267	*P* = .787	*P* = .134	*P* = .346	*P* = .391
Displacement	*P* = .198	*P* = .880	*P* = .061	*P* = .350	*P* = .155	*P* = .397
Intra-articular involvement	*P* = .662	*P* = 1.0	*P* = .662	*P* = .087	*P* = .565	*P* = .156
No. of fragments	*P* = .851	*P* = .983	*P* = .662	*P* = .950	*P* = .723	*P* = .662

Abbreviations: L&B, Lawrence and Botte classification; MCS, Mental Component Summary; PCS, Physical Component Summary; SF-12, quality-of-life score; VAS FA, visual analog scale for foot and ankle; Δ, patient-reported outcome measure difference between 2- and 5-year follow-up.

## Discussion

Studies on functional treatment for Lawrence and Botte type I and any type II 5th MT fractures are sparse and limited to short-term results only. This is the first study reporting longer-term results for these fractures. Furthermore, it is among few analyzing the influence of the fracture characteristics on the patient-rated outcome. Overall, patients achieved an excellent foot and ankle function (VAS-FA) and quality of life (SF-12) after a median follow-up of 5.7 (1.6) years following functional treatment. Neither the fracture location (type I vs II) nor the fracture characteristics (displacement, intra-articular involvement, number of fragments) had an influence on the outcome. These promising results were also observed in patients with a longitudinal follow-up after 2 and 5 years. No deterioration of the excellent results was observed over time.

Studies on type I fractures, including an analysis of the fracture characteristics and correlation of the clinical results, are sparse.^9,29,33^ Egol et al^9^ treated 50 type I fractures (50% intra-articular, 32% displaced >2 mm) by immediate weightbearing. None of the fracture characteristics (intra-articular involvement or displacement) had a significant influence on the Short Musculoskeletal Function Assessment or the VAS for pain after 1 year. Tahririan et al^29^ reported the patient-reported outcome (American Orthopaedic Foot & Ankle Society [AOFAS]) after 20 weeks in patients with type I, II, and III fractures all treated by nonweightbearing in a short leg cast for 8 weeks. Based on a multivariate analysis, factors influencing the AOFAS were displacement, patient weight, type III fractures, diabetes, and female sex. The 2 most pronounced limitations of this study were the short follow-up and the lack of detailed statistics. As the AOFAS score for all fractures was excellent (93; 95% confidence interval: 92-94), it remains particularly questionable whether these differences were of any clinical relevance. Finally, 1 randomized controlled trial compared percutaneous screw fixation to nonoperative treatment in displaced (2- to 3-mm) type I fractures.^33^ No significant differences were observed for the AOFAS after 12 months except a significantly shorter time to return to work for the operative cohort (8.1 ± 0.9 vs 9.3 ± 1.0 weeks; *P* < .05). Unfortunately, the nonoperative group was treated by 6 weeks of immobilization and nonweightbearing, which has been proven inferior compared to functional treatment^1^ and full weightbearing.^26^ In line with these findings, the herein reported functional treatment regimen resulted in a significantly shorter time to return to work (mean, 2.5 weeks) and sports (mean, 7.8 weeks) than for any type I fracture results published previously.^3^ The current study strongly supports functional treatment for all type I fractures, independent of displacement, articular involvement, and comminution.

For type II fractures, most authors recommend either nonweightbearing and immobilization for 6 to 8 weeks or operative management.^8,16,19,20^ Few have suggested functional treatment.^6,9,15^ Without much question, a consistent issue with these injuries is simply one of nomenclature, as these fractures are called by many names, some of which are not always clear in terms of what they represent. In particular, the eponymic term *Jones fracture* has been used inconsistently to describe many of these injuries (both types II and III), although it is a very specific injury and ultimately represents only a small proportion of all 5th MT base injuries.^2,22^ Furthermore, frequently new variations of this eponym are being introduced. To give one example, a recent systematic review comparing the outcomes after treatment with either a short-leg cast or a splint, the authors included exclusively “pseudo-Jones avulsion fractures.”^21^ Unfortunately, it remains elusive which fracture types have been included, and therefore the therapeutic consequence of such studies remains limited. To clearly define the exact fracture location is of great importance as type III fractures, the proximal diaphyseal injuries, are at greatest risk for delayed union and nonunion. Consequently, the term *Jones* *fracture* should be avoided as it does not clearly define the fracture location.

When looking at studies that included actual type II fractures, types I and II apparently behave similar in terms of prognosis following nonoperative treatment.^12,30,32^ This observation is strongly supported by the findings presented in this study. Functional treatment—namely, immediate free range of motion and full weightbearing—led to excellent 5-year results for both type I and II fractures, with no significant differences between the 2 fracture locations. It might be true that the worry with respect to healing of type II fractures has perhaps been overstated, and consequently, type I and II fractures can be summarized as epi-metaphyseal. Various factors such as sex, age, body mass index, and diabetes mellitus have been reported to affect the outcome following nonoperative treatment of type I and II fractures.^6,7,29,31^ However, these findings have been inconsistent and contradictory between studies. In the current cohort, neither age nor sex had a significant influence on the 5-year outcome.

The authors are aware of only 2 studies providing a follow-up of more than 2 years: one with 2.2 years^19^ and the other with 2.7 years.^15^ Therefore, this study provides the longest follow-up reporting objective measures. Despite the current excellent results after 5.7 (1.6) years for both type I and II fractures, one could argue that the follow-up period provided is still too short to identify symptomatic posttraumatic osteoarthritis. Interestingly, symptomatic osteoarthritis of the fifth tarsometatarsal (TMT) joint overall is a very rare condition.^24^ This is notable as the incidence of fractures of the base of the 5th MT has been reported as high as 1.8 per 1000 person years.^27^ If they would regularly lead to symptomatic osteoarthritis, one would expect to observe this condition more frequently. Little is known about the incidence and the causes, but it seems to develop most commonly secondary to malalignment of the hind- or midfoot, inflammatory disease, or to simply be idiopathic.^5^ Posttraumatic osteoarthritis seems to be caused primarily by injuries to the lateral column such as fracture of the cuboid or Lisfranc injuries.^25^

There are several limitations of this study. The study design used a retrospective data assessment. However, this study resembles a natural history study and is based on a prevalent cohort that did not receive an intervention.^13^ Second, the final follow-up rate was limited to 45%. However, this is similar to the follow-up in many previous comparable studies.^1,17^ Due to the retrospective nature of the study, no a priori power analysis could be conducted. The value of post hoc power analysis is under heavy dabate and therefore was not conducted. Another limitation could be the missing radiographic follow-up. We do not routinely conduct radiographic follow-ups. A radiographic control is performed only in case of prolonged symptoms of more than 6 weeks.^10^ The rationale behind this procedure is that asymptomatic nonunions or asymptomatic osteoarthritis have no consequence of treatment. Finally, the follow-up period of 5.7 years might still be too short to detect symptomatic osteoarthritis, yet up to now, it is the longest follow-up period available.

In conclusion, the data presented strongly suggest that Lawrence and Botte type I and II 5th MT base fractures may be better termed as epi-metaphyseal fractures as they share the same excellent prognosis. Furthermore, based on the data available, the above outlined fracture characteristics apparently do not predispose to symptomatic osteoarthritis. Consequently, any 5th MT epi-metaphyseal fracture, independent of its fracture characteristics (ie, displacement, articular involvement, and number of fragments) can be treated functionally with unrestricted full weightbearing.

## Supplemental Material

FAI907391_disclosure – Supplemental material for Results of Functional Treatment of Epi-Metaphyseal Fractures of the Base of the Fifth Metatarsal Click here for additional data file. Supplemental material, FAI907391_disclosure for Results of Functional Treatment of Epi-Metaphyseal Fractures of the Base of the Fifth Metatarsal by Sebastian F. Baumbach, Marcel Urresti-Gundlach, Wolfgang Böcker, J. Turner Vosseller and Hans Polzer in Foot & Ankle International
